# Phase contrast flow imaging in Cardiac Magnetic Resonance (CMR): Imaging turbulent blood flow

**DOI:** 10.1186/1532-429X-17-S1-T3

**Published:** 2015-02-03

**Authors:** Chris B Lawton, Stephen Lyen, Jonathan C Rodrigues, Nathan E Manghat, Chiara Bucciarelli-Ducci, Mark Hamilton

**Affiliations:** 1 CARDIAC MRI UNIT, Brstol Heart Institute, Bristol, UK

## Background

Accurately measuring blood flow volume is paramount in patients with arterial valve disease. Flow measurements are important components of a CMR report and provide the referring clinicians with valuable information from which to base further clinical management. There are many factors which influence the quality and reliability of flow data including; slice positioning, velocity encoding mismatching, temporal resolution, and turbulence.

**Figure 1:**
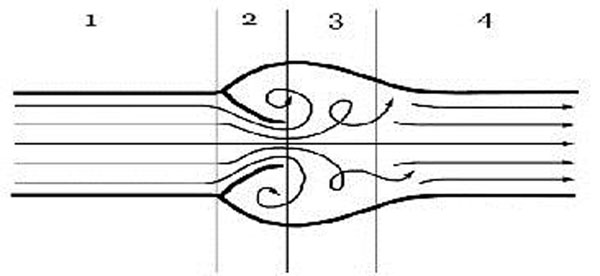
**Diagram of flow across a semilunar valve.** 1. Laminar flow through the proximal vessel. 2. The transient reduction in diameter as the blood flows across the valve results in increase in velocity due to the Bernoulli principle. 3. The high velocity results in post valvular turbulence. Signal dropout occurs in this region due to the disorganised flow. 4. Eventually at a distance from the valve, laminar flow resumes. When there is regurgitation the turbulence may also be seen in a subvalvar position.

Turbulence can be caused when vavular pathology is present. Blood flowing through the valve will become non laminar, and eddies and swirls which are produced can lead to signal dropout. (Figure 1).

## Methods

**Figure 2:**
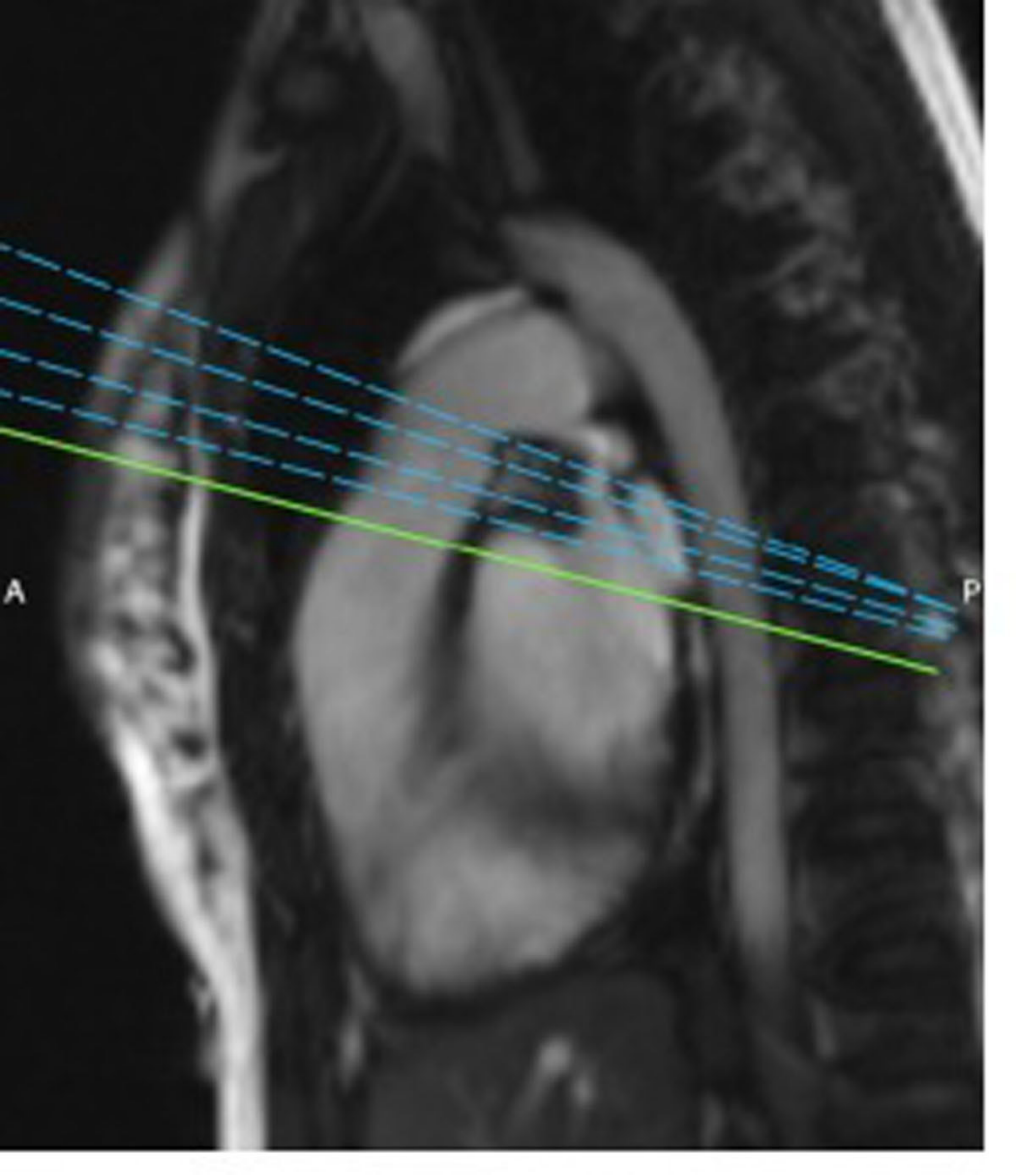


A retrospective study conducted at our centre analysed vavular flow reports in 25 patients with pulmonary valve (PV) assessment from Sept 2011 - present. A stack of measurements ranging from 3-6 slices were taken across the PV (Figure 2), and the following data recorded:

• Antegrade and retrograde flow volumes, Anatomical slice positions

• Degree of antegrade/retrograde turbulence

• Variation in antegrade flow = Max - Min antegrade flow volume

• Variation in regurgitant fraction = Max- Min calculated regurgitant fraction

• The ratio of variation in flow volume to the mean antegrade flow for each patient was calculated.

## Results

The mean antegrade flow volume variation was 11.2 ml (13.1% of mean antegrade flow).

Mean retrograde flow volume variation was 7.1 ml (33% of mean retrograde flow).

The mean variation in regurgitant fraction was 7.4%.

## Conclusions

These results show a significant variation in estimated antegrade and retrograde flow volume depending on the position across the valve at which the data was acquired. This would have a significant impact on any subsequent interventional decisions.

When turbulent flow is present no single slice position can be assumed to give an accurate result. Typically flow distal to the valve underestimates antegrade flow, and retrograde flow is underestimated proximal to the valve. Assuming the maximum measured antegrade and retrograde flow is closest to the true flow it would be appear to be more accurate to obtain a stack of phase contrast images through the valve to establish the true antegrade and retrograde values, and therefore better define the regurgitation fraction

## Funding

National Institute of Health Research, Biomedical Research Unit.

